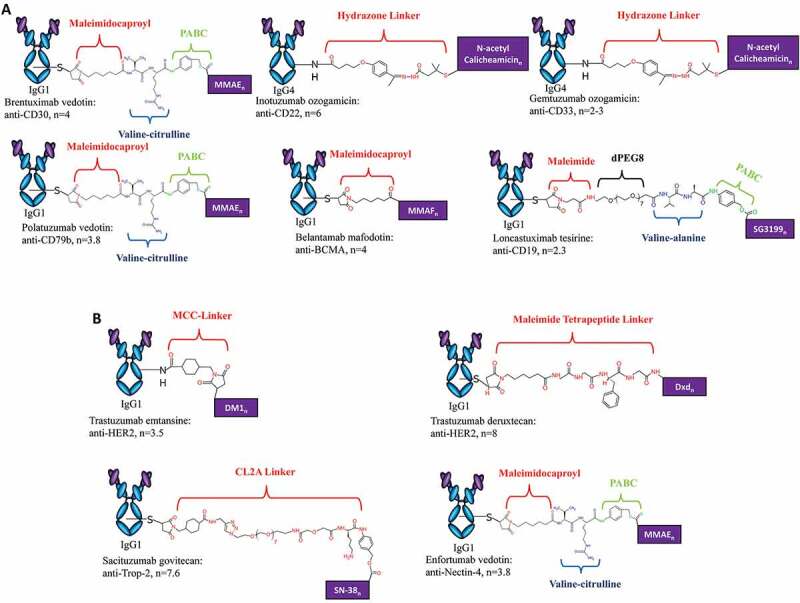# Correction

**DOI:** 10.1080/19420862.2021.1966993

**Published:** 2021-08-25

**Article title**: Targeting cancer with antibody-drug conjugates: Promises and challenges

**Authors**: Alexis Q. Dean, Shen Luo, Julianne D. Twomey, and Baolin Zhang

**Journal**: *mAbs*

**Bibliometrics**: Volume 13, 2021, Issue 1

**DOI**: https://doi.org/10.1080/19420862.2021.1951427

It has been noted by the authors that the Figures 2 was published with errors. These errors have now been corrected as shown below. This correction has not changed the description, interpretation, or the original conclusions of the article. The authors apologize for any inconvenience caused.

**Figure 2.**